# Exploring the group heterogeneity in the impact of social cohesion on the walking frequency of older adults in China

**DOI:** 10.3389/fpubh.2024.1424975

**Published:** 2024-07-31

**Authors:** Jingwen Ma, Wu Li

**Affiliations:** ^1^School of Public Administration, Dalian University of Technology, Dalian, China; ^2^Faculty of Transportation Engineering, Kunming University of Science and Technology, Kunming, China

**Keywords:** perceived walkability, aging, body mass index (BMI), multiple-group analysis, built environment, structural equation model (SEM)

## Abstract

**Background:**

Walkable neighborhoods are closely related to an increase in walking frequency and the strengthening of social cohesion. These factors, in turn, contribute to lower BMI and other positive health-related outcomes. However, with a rapid increase in aging populations in China and the fact that women are facing more challenges than men as they age, especially mobility challenges because they tend to live longer leading to probabilities to become widowed. Nevertheless, less attention has been paid to understanding the gender difference between these relationships.

**Methods:**

Based on a survey of 533 older adults in Dalian, China, this study tried to investigate the intertwined relationship between perceived walkability, social cohesion, walking frequency, and BMI. A Structural Equation Model (SEM) and multiple-group analysis were applied to test the proposed framework.

**Results:**

First, results show that gender differences existed among the above interrelationships, and the most substantial gender gap was found in effects of social cohesion on BMI. Second, perceived walkability only has a direct effect (0.149) on walking frequencies for female seniors. Third, although the relationships between perceived walkability and BMI are not directly related in both male and female models, the indirect connection (−0.053) is substituted for female seniors. Besides, the inhibiting effect of walking on BMI, which is −0.511, is also valid for female seniors. Finally, in terms of the role of social cohesion, both the positive impacts of perceived walkability on it (0.225 for males and 0.325 for females) and its promoting effects on walking have been confirmed in male (0.142) and female models (0.103). The negative direct effect of social cohesion on BMI (−0.083) is only confirmed in male seniors.

**Conclusion:**

Insights derived from this analysis can help bring forward gender-specific interventions to build a more inclusive walkable and social environment to improve the mobility and physical health of older adults.

## 1 Introduction

Worldwide, demographic aging has gradually become a prevalent social phenomenon due to the sharp decline in fertility and the gradual increase in average life expectancy, China is not an exception. According to the seventh population census of China, the proportion of people aged over 60 is 18.7%, and women is 9.3 million more than men among those old people by the end of 2020 in China (1). Women generally outlive men, leading to a growing gender gap with age (2). For example, in China in 2020, there were 13.5 women over 80 for every 10 men of the same age (1). As a result, older women are more prone to mobility issues in their later years. This is often due to living alone or becoming widowed (2). Furthermore, the same influencing factors may also have different effects on the physical activities or travel behavior of males and females due to their different domestic and labor roles (2, 3). For example, research stated that greater social cohesion was related to adequate physical activity only in men (3). Additionally, a previous study found that medical conditions had a higher impact on the mobility of female older adults (2). Mobility and social engagement are the basic prerequisites to healthy aging (4). Involvement in activities outside the home has been associated with better health status (5). However, it is concerning that, as indicated by the 2020 National Physical Fitness Surveillance, compared to the 2014 data, there has been a significant increase in the average levels of weight, waist circumference, and hip circumference among the seniors in China, with a notable rise in the rate of obesity. In 2020, the overweight and obesity rates among adults were 35.00 and 14.60%, respectively, while among the seniors, the overweight and obesity rates were 41.70 and 16.70%, respectively. This highlights the growing challenge of obesity among the older adults, which is critical to consider in the context of promoting health and well-being through social integration and physical activity (6). As the primary travel mode for seniors to participate in social life and run errands in cities in China, walking has been confirmed could produce considerable health benefits in reducing the risks of obesity and other related chronic diseases (7, 8), particularly for women (9). However, this, coupled with the fact that the greater challenges faced by women as they age, has made the execution of gender-specific interventions to improve social integration and wellbeing become urgent for older adults (2, 5).

A plethora of research has examined the relationships between the built environment, walking frequency, and BMI (10–12). The built environment is an important contributor to both promoting walking and alleviating body weight (7). Evidence shows that walking is beneficial for reducing the risk of being overweight (13). However, previous studies have not adequately addressed the impact of social cohesion on walking frequency and BMI. Research has confirmed that higher social inclusion, which could be achieved by participating in social activities and joining neighborhood services, was connected with more walking trips and better health status (5, 14, 15). For better understanding, it is important to incorporate social cohesion into the relationship between the built environment, walking frequency, and BMI. This is relevant to the efficacy of planners and policymakers to develop prosocial policies to promote walking, and ultimately, maintain good physical health. However, considering the gender imbalance in mobility, transportation, urban health, etc., it is necessary to identify the differentiated nature between female older adults and male older adults when conducting such an analysis (2). This will help planners to make gender-specific interventions rather than treating older adults as a single group so that they can frame plausible planning decisions targeting the unique needs of a different gender to improve their walking-related built and social environment, thereby promoting more health-related travel activities. Furthermore, fewer studies have focused on detecting the above-mentioned relationships in rapidly urbanizing developing countries like China, with only a few studies studying such interrelations using data collected in various metropolitan city, i.e., first-tier city, like Hong Kong (5). It is still uncertain whether generalizations made about China’s first-tier cities apply to second-tier cities, given that the quality, planning, and construction of neighborhood built environment might be different from one place to another, which might further provide different opportunities and motivations for walking. In the context of China’s urban hierarchy, second-tier cities are major provincial capitals and large cities that, while not as globally recognized as first-tier cities like Beijing and Shanghai, still hold significant economic and demographic importance. These cities are characterized by a population size that typically ranges from several million to over 10 million inhabitants. For example, Dalian, which is the focus of our study, are significant provincial capitals with substantial economic and demographic importance. Dalian, with a population of approximately 7.4507 million according to the latest census (1), exemplifies a substantial urban center. This classification is crucial for understanding the unique social, economic, and environmental factors influencing the lifestyle and health behaviors of its residents. First-tier cities in China, often recognized as the largest and most prosperous, boast a highly developed walkability environment as measured by the Neighborhood Environment Walkability Scale (NEWS). These megapolises are characterized by extensive and well-maintained pedestrian infrastructure, high accessibility to a variety of destinations such as commercial hubs, cultural venues, and educational institutions, and a strong emphasis on pedestrian safety with thoughtful traffic designs. Moreover, their esthetic appeal is enhanced by quality green spaces, landscape designs, and modern, attractive architecture. In contrast, second-tier cities, which are typically medium-sized with a solid foundation and robust business activity, may not match the first-tier cities in terms of walkability. While they still offer basic pedestrian facilities and a certain level of destination accessibility, particularly in city centers, they might have some areas that require improvement in terms of safety and esthetic appeal. These cities, however, often possess unique cultural and historical features that contribute to their walkability environment, albeit at a different scale and with different priorities compared to the first-tier cities. Conducting such an analysis focusing on a second-tier city like our study area, Dalian, might help provide targeted general planning policies to other similar cities on how to improve walkability and other health-related environments.

This research attempted to answer the following questions: (1) How built environment, walking frequency, and BMI are related to each other? (2) What are the effects of social cohesion on the interrelations mentioned in the question (1)? (3) Is there a significant difference existing between male older adults and female older adults? By applying a Structural Equation Model (SEM) and multiple-group analysis, this study tried to estimate the impact of gender on different measures of the BMI of older adults by analyzing data collected on 533 older adults aged 60 in October 2021 in Dalian, China.

The remainder of this paper is organized as follows: Section 2 introduces the theoretical backgrounds and proposed the conceptual framework of this study. Section 3 describes the data and methodology, which is followed by the results in Section 4. Section 5 describes the discussion of this research. Finally, the major conclusion and was summarized in Section 6.

## 2 Theoretical backgrounds

### 2.1 Built environment, walking frequency, and BMI

The impact of the built environment on walking frequency has attracted a lot of attention in the field of transportation research, and both the objective and perceived measures of the built environment are considered as key contributors to walking (10, 11, 16). The 5Ds model is a widely recognized framework for assessing the objective aspects of the built environment (17). It encompasses five key dimensions: density, diversity, design, destination accessibility, and distance to public transportation. Research addressing those “5Ds” has been confirmed to promote walking frequency. For example, first-tier cities, often megapolises, exhibit high density with a concentration of services and amenities, a diverse mix of land uses, well-planned and esthetically pleasing urban designs, high accessibility to essential destinations, and close proximity to public transportation networks, all of which promote walking. In contrast, second-tier cities, while not as densely populated, still provide a range of land uses and services that contribute to their walkability, albeit on a smaller scale. They may have areas that require improvement in design and accessibility but are actively working toward enhancing their urban environments to encourage walking (10, 16). However, the cognitive feeling of the same neighborhood might differ between individuals even if the objective measures are believed to capture a less fluctuating view of the environment (18). Also, cognitive perceptions of the environment are confirmed as more close to health-related behavior and health (18, 19). The perceived measures are usually obtained from self-reported questionnaires, which would reflect their subjective evaluation of neighborhoods. To examine its effects on walking frequency, a variety of indices has been applied to measure “perceived walkability” (20–22), the NEWS and its derivatives are the most commonly used indicators (20). A more walkable environment, which includes a clean sidewalk, comfortable weather, better-perceived accessibility, etc., has an association with increasing walking frequency (11, 18). There is a lack of consensus on which kind of built environment should be included to examine the potential effects on walking frequency. Research has shown that the perceived measure had a better performance when examining the impacts on travel behavior (23). Thus, we propose that:

H1: The perceived walkability is positively associated with walking frequency.

Body mass index is a common indicator to measure obesity and overweight, and both the built environment and walking frequency are determinants of BMI (7, 13, 23). Although some researchers have found no evidence showing a connection between the built environment and BMI (24, 25), most existing studies have found that the built environment is a factor related to weight loss (23, 26). For example, Walkability indexes, used to gauge the perceived built environment, have revealed that people aged 45 in Sydney who live in areas with high walkability are less likely to suffer from obesity (27). Furthermore, a burgeoning body of research has demonstrated that people frequently walking for different purposes could potentially help reduce their BMI (13, 24, 27). Based on the above findings, we hypothesize the following:

H2a: Perceived walkability has a negative correlation with BMI.

H2b: Walking frequency is associated with the reduction of BMI.

### 2.2 The role of social cohesion

Social cohesion could be defined as the degree to which a neighborhood is socially interrelated, i.e., the extent to which residents feel they belong to their neighborhoods (15). It could reflect people’s shared norms, feeling of trust, and connectivity with their neighborhoods (28). Social cohesion is usually measured by questions on the Likert scale to realize a neighborhood’s sense of belonging, friendliness, connectedness, and sociability (5, 15, 29). It is a popular topic in both transportation and health-related research (28–31). The effects of perceived social cohesion on several social outcomes that include a positive change in health behavior, more participation in physical activity, and a stronger sense of neighborhood attachment have been substantiated (15, 28, 30). Living in a perceived walkable neighborhood, residents have more opportunities to unexpectedly interacted with neighbors and have a catalytic effect on developing their comfort, empowerment, and sense of cohesion (29, 32). Besides, the objective built environment, mixed land use, sidewalk prevalence, and lower prevalence of heavy traffic, etc., was associated with a stronger sense of social involvement and cohesion (28, 33).

Past research has consistently found that social cohesion increases walking behavior (15, 31, 34). For example, evidence found seniors who live in a more cohesive community are more willing to increase their walk frequency to exercise (31). Work by Moreover, findings indicate that residents who feel a stronger sense of cohesion in the neighborhoods spend more time walking (15).

The relationships between social cohesion and BMI are complex. One study found a positive relationship existed between them (25), another found no significant association (35), while the majority of studies demonstrated that a negative relationship (24, 36). Participation in a geographic neighborhood context makes people feel more emotionally related and can be provided enough social support, which will help decrease the risk of depression, and in turn, lower the residents’ risks of becoming overweight (5, 36). Taking up the positive role of social cohesion, the following hypotheses were proposed:

H3a: Perceived walkability has a positive connection with social cohesion.

H3b: Social cohesion is positively related to walking frequency.

H3c: Social cohesion is negatively associated with BMI.

**Figure 1:**
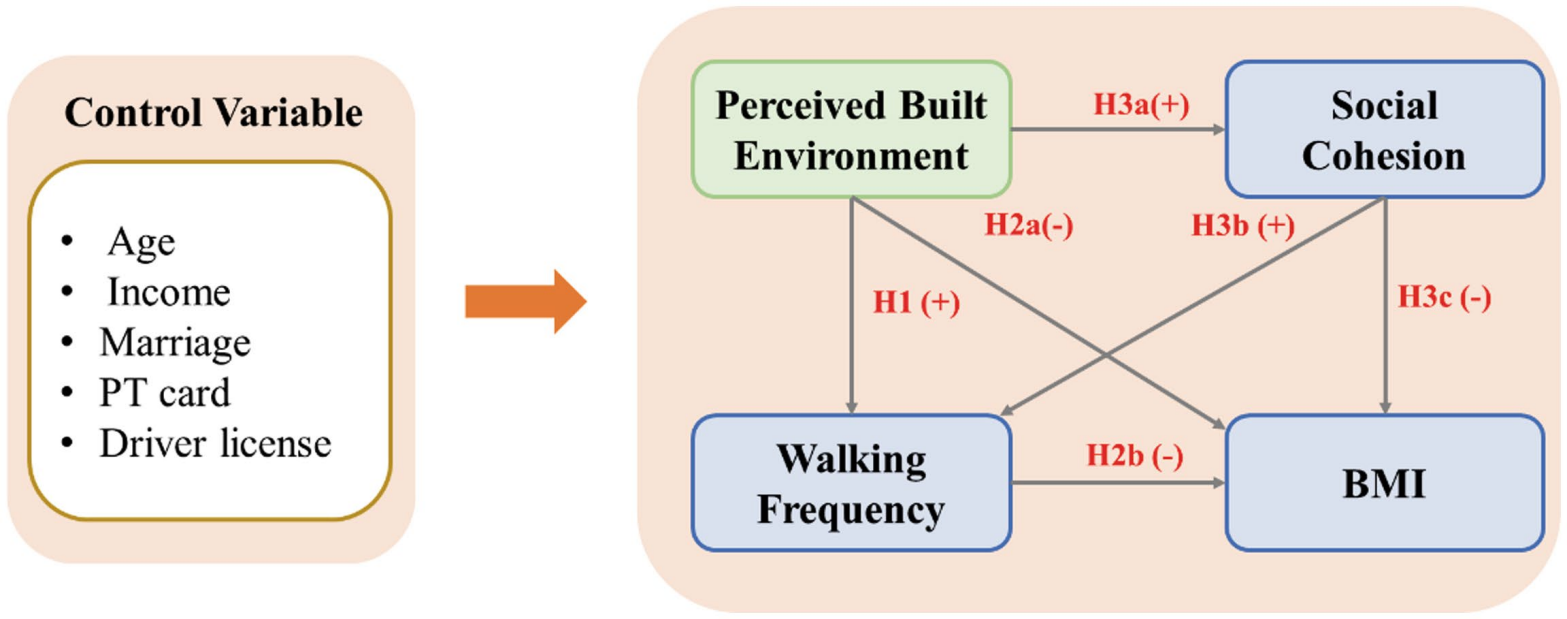
Conceptual framework.

The conceptual framework presented in Figure 1 illustrates the complex interplay between several key variables that influence walking frequency and BMI. At the core of this framework are the perceived built environment, social cohesion, walking frequency, and BMI, which are the primary constructs of interest in this study. The conceptual framework includes the three hypotheses that we mentioned before. In addition to these central variables, the framework incorporates a set of control variables that may have an impact on the relationships being explored. To be specific, age, income level, marriage status, driver license ownership, and a concessionary pass for public transportation ownership (PT card) were considered. By involving those control variables, it helps us to capture the potential correlations more precisely.

## 3 Data and methodology

### 3.1 Data collection

The study was conducted via face-to-face interviews from 18 October 2021 to 30 October 2021 in Dalian City, Liaoning Province, China. Given that Dalian’s urban area comprises only four main districts—Zhongshan District, Shahekou District, Xigang District, and Ganjingzi District—our study focused on these districts to ensure a comprehensive urban representation.

To select participants, we targeted residents aged over 60, following the current retirement age criteria in China, where women retire at 50 or 55 and men at 60. We employed a convenience sampling method to recruit participants randomly across the four districts. To ensure a diverse and representative sample, we approached potential participants in various public locations such as parks, local squares, senior centers, and streets.

Participants were included based on the following criteria: (1) at least 60 years old; (2) having lived in the current neighborhood for at least 6 months; (3) having no cognitive impairment; and (4) having the ability to walk.

Eight surveyors from Dalian University of Technology were recruited and sufficiently trained before the surveys to avoid mistakes. The investigators initially informed the potential participants of their rights, the purpose of this study, the procedures to be performed, and the potential risks and benefits of participating in this study. Oral information about answer confidentiality and safe data keeping was given to each participant. Additionally, they were given the assurance of anonymity and reminded that participation was entirely voluntary.

Anonymous investigations that do not involve minors are exempt from formal ethical reviews according to Chinese ethical research standards. However, we consulted the officer from the ethics committee from our university to make sure that the methodological process met good ethical standards. Those who are eligible and agree to participate finished the questionnaire with the help of surveyors.

For the purpose of this study, our team reached out to a total of 618 older adults, 533 of them responded to the survey with full information, complete records, careful answers, etc. The response rate of our study is 86.25%.

### 3.2 Measurements and descriptive statistics

#### 3.2.1 Perceived walkability

The Neighborhood Environment Walkability Scale (NEWS) proposed by and its abbreviated version (NEWS-A) developed by are the normal measures of perceived walkability (20, 21, 37–39). Chinese Walkable Environment for the urban community (40), which stems from NEWS-A, was used to measure perceived walkability in this study. It is chosen because its reliability (Cronbach’s alpha = 0.807) was examined to be good, and its factor validity was further supported with confirmatory factor analysis (40). 13 items from four aspects of the perceived walkability, i.e., accessibility, road condition, esthetics, and safety, were derived from the confirmatory factor analysis (CFA) to measure perceived walkability in this study (Table 1). An example of the item was “*It is easy to go to* var*ious places from my house in my neighborhood*.” Participants reported their level of agreement with each question on a five-point Likert scale ranging from “strongly disagree” to “strongly agree.” This scale showed good internal consistency for full sample (Cronbach’s α = 0.878), male seniors (Cronbach’s α = 0.891), and female seniors (Cronbach’s α = 0.862).

**Table 1:** Descriptions and summary statistics of variables (*N* = 533).

Variable	Description	Mean/Count	SD/%
Perceived walkability			
Accessibility (AC1)	It is easy to walk from my house to the shops.	3.78	0.82
(AC2)	It is easy to walk to a transit stop from my home.	3.87	0.75
(AC3)	It is easy to go to various places from my house in my neighborhood.	3.66	0.76
Road Condition (RC1)	The road around my house is in good condition in terms of greenery.	3.47	0.90
(RC2)	The roads are clean and tidy in my neighborhood.	3.48	0.89
(RC3)	My neighborhood streets are well lit at night.	3.60	0.88
(RC4)	The streets in my neighborhood are flat.	3.39	1.08
Esthetics (AE1)	There are enough places around my house exercise.	3.30	0.96
(AE2)	The natural environment around my home is good, which makes me want to go out and walk.	2.87	1.08
(AE3)	The buildings around my house are very attractive.	2.95	0.97
Safety (SA1)	My neighborhood is very safe.	3.79	0.84
(SA2)	My neighborhood is very safe during the day.	3.90	0.77
(SA3)	My neighborhood is very safe at night.	3.87	0.79
Social cohesion			
Mutual help (MH)	Residents in my neighborhood are willing to help their neighbors	3.63	0.86
Connection (CN)	I live in a tight-knit community.	3.42	0.92
Trust (TR)	The residents in this community are trustworthy.	3.45	0.89
Acquaintanceship (AC)	I know most of my neighbors.	3.16	1.00
Walking frequency	Walking frequency in the last seven days	23.66	13.97
BMI	Underweight	20	3.75
	Normal	187	35.08
	Obesity	240	45.03
	Overweight	86	16.14
Socio-demographic variables (Control)			
Age	60–64	121	22.70
	65–69	146	27.39
	70–74	105	19.70
	75–79	75	14.07
	Over 80	86	16.14
Gender	Male	227	42.59
	Female	306	57.41
Income	<50,000 RMB/year	133	24.95
	50,000–100,000 RMB/year	266	49.91
	>100,000 RMB/year	134	25.14
Marriage	Widowed or divorced	90	16.89
	Married	443	83.11
Public transportation concessionary pass ownership (PT card)	No	78	14.63
	Yes	455	85.37
Driver license ownership	No	467	87.62
	Yes	66	12.38

#### 3.2.2 Walking frequency

The respondents were requested to recall their walking experience in the last 7 days and they were asked to report the total number of walking trips over 10 min. As shown in Table 1, the average number of walking trips of older adults is 24 times per week.

#### 3.2.3 Body mass index

Body mass index is a common measurement of weight to decide whether a person is underweight, normal weight, overweight, or obese. It is calculated by dividing the participant’s weight in kilograms by the square of their height in meters. According to the standard by the Chinese Ministry of Health, a BMI below 18.5 is considered underweight, the value of it between 18.5 and 24 is defined as normal weight, when it is between 24 and 28 is considered overweight, and it is treated as obese when exceeding 28. Participants were further grouped based on this division. Among respondents in our study, 45.03% of them are overweight, and 16.14% of them are obese (Table 1), the prevalence of overweight and obesity among older adults in China is approximately 41.70 and 16.70%, respectively (6). The slightly higher rates of overweight and obesity in our sample compared to the general adult population underscore the need for targeted health interventions for older adults.

#### 3.2.4 Social cohesion

Four aspects of neighborhood cohesion—mutual help, connection, trust, and acquaintanceship—were measured (41). Subsequently, we used four items of the scale rated on a 0–5 Likert scale (Table 1). A higher score on these items indicates a strong sense of perceived trust and social cohesion. An example of the description of those items was “*The residents who live in this community are trustworthy*.” Cronbach’s alpha for this scale was 0.872, 0.877, and 0.867 for the full sample, male older adults, and female older adults, respectively, which indicates good internal consistency.

#### 3.2.5 Socio-demographic variables

Specifically, the following socio-demographic variables were involved: age, gender, household income level, marriage status, driver license ownership, and a concessionary pass for public transportation ownership (PT card). The descriptive statistics of socio-demographic variables are shown in Table 1. The results show that the distribution of the samples is almost equal across different age groups. However, older adults between 60 and 64 (22.70%) and between 65 and 70 (27.39%) are slightly higher than in the other groups. The percentage of females (57.41%) is slightly higher than that of males (42.59%). Among respondents, the vast majority of them have a public transportation concessionary pass, while the possession of driver license is exactly the opposite, i.e., most of them failed to have a driver license.

### 3.3 Methods

A structural equation model (SEM) is utilized to examine the underlying relationships between the latent constructs. It is composed of two fundamental models: a measurement model and a structural model. The measurement model is a linear model of the latent constructs as a function of observed indicators, and the structural model reflects the interrelationships between latent constructs (42).

We followed the dominant approach in the literature to sequentially examine the measurement model and structural model instead of estimating them simultaneously in one-step (42). The measurement model adequacy was assessed by Confirmatory Factor Analysis (CFA) before exploring the structural relationships.

A moderator is a variable that alters the intensity and direction of the relationship between dependent variables and independent variables (43). Multiple-group SEM is a widely used method to examine whether considerable differences exist among different sample groups defined by a categorical moderator (sex and race, etc.) (42). Hence, multiple-group SEM is applied to investigate if and how much the relationships between perceived walkability, social cohesion, walking frequency, and BMI significantly varies across the gender of older adults.

The approach to multiple-group comparisons involves a hierarchical evaluation that imposes increasingly stringent constraints on the model (44). First, *configural invariance* intends to examine the fit of the model for male and female seniors independently with unrestricted conditions by evaluating each group with the same measurement and structural model. Following this, *weak in*var*iance* was tested by increasing the restriction of the factor loadings to remain the same in each group. Then, both the factor loadings and the intercepts of the measurement models were fixed for both groups, i.e., *strong in*var*iance*. Finally, *strict invariance* refers to that factor loadings, measurement intercepts, and measure residual variances are equal among different groups. The null hypothesis points out that the groups, i.e., male seniors and female seniors, have equivalent factor variance. In other words, the rejection of this null hypothesis indicates that the factors are not invariant and groups influence the causal structural relationship (45).

Chi-square differences tests were chosen to evaluate invariance in this study, a significant value of Chi-square indicates that the null hypothesis should be rejected (46). Stata 17 software was used to perform all the computations in this study.

## 4 Results

### 4.1 Measurement model

Confirmative factor analysis (CFA) was performed to assess fit indices and parameter loadings of the proposed model. As can be seen from Table 2, all the loadings in the models are statistically significant in their respective constructs. The Composite Reliability (CR) and the Average Variance Extracted (AVE) values were calculated to confirm the reliability of our measurement model. The values of CR range from 0.775 to 0.890 for the full sample, they range from 0.774 to 0.909 for models of male seniors, and they range from 0.759 to 0.886 for the model of female seniors, which are both greater than 0.7 (42). Both these three models have acceptable values of AVE, which is equal to or greater than 0.5 (42). Finally, according to Hu and Bentler (47), an ideal fit model has the values of CFI and TLI equal to or greater than 0.90, and SRMR equal to or less than 0.08. The values of SRMR of all three models are 0.061, 0.075, and 0.062, respectively. Furthermore, both the values of CFI and TLI are greater than 0.90 indicating a good model fit.

**Table 2:** Measurement model results.

	Full			Male			Female		
Construct	Loads	S.L.	*p* value	Loads	S.L.	*p* value	Loads	S.L.	*p* value
Perceived walkability									
*Accessibility*									
AC1	1.000	0.713	–	1.000	0.771	–	1.000	0.667	–
AC2	1.010	0.786	^***^	0.871	0.800	^***^	1.179	0.790	^***^
AC3	0.900	0.693	^***^	0.799	0.679	^***^	0.992	0.688	^***^
***CR***	0.775			0.775			0.759		
***AVE***	0.536			0.536			0.514		
*Road condition*									
RC1	1.000	0.837	–	1.000	0.867	–	1.000	0.797	–
RC2	1.022	0.861	^***^	1.023	0.892	^***^	1.029	0.827	^***^
RC3	0.809	0.701	^***^	0.776	0.744	^***^	0.862	0.669	^***^
RC4	0.839	0.582	^***^	0.814	0.563	^***^	0.920	0.623	^***^
***CR***	0.838			0.856			0.828		
***AVE***	0.568			0.605			0.549		
*Esthetics*									
AE1	1.000	0.748	–	1.000	0.761	–	1.000	0.731	–
AE2	1.178	0.783	^***^	1.165	0.785	^***^	1.195	0.777	^***^
AE3	0.961	0.711	^***^	0.841	0.639	^***^	1.071	0.771	^***^
***CR***				0.774			0.784		
***AVE***				0.535			0.548		
*Safety*									
SA1	1.000	0.818	–	1.000	0.820	–	1.000	0.814	–
SA2	1.007	0.896	^***^	1.018	0.920	^***^	0.996	0.875	^***^
SA3	1.010	0.876	^***^	1.007	0.890	^***^	1.010	0.861	^***^
***CR***	0.890			0.909			0.886		
***AVE***	0.729			0.770			0.723		
Perceived walkability									
*Accessibility*	1.000	0.545	–	1.000	0.534	–	1.000	0.528	–
*Road condition*	2.005	0.856	^***^	2.000	0.862	^***^	2.070	0.848	^***^
*Esthetics*	1.858	0.828	^***^	1.918	0.905	^***^	1.853	0.743	^***^
*Safety*	1.264	0.588	^***^	1.274	0.625	^***^	1.288	0.535	^***^
***CR***	0.803			0.829			0.765		
***AVE***	0.515			0.560			0.500		
Social cohesion									
MH	1.000	0.803	–	1.000	0.777	–	1.000	0.853	–
CN	1.148	0.870	^***^	1.169	0.848	^***^	1.075	0.885	^***^
TR	1.087	0.844	^***^	1.200	0.900	^***^	0.946	0.792	^***^
AC	0.974	0.676	^***^	1.069	0.702	^***^	0.869	0.654	^***^
***CR***	0.877			0.883			0.876		
***AVE***	0.643			0.656			0.642		
***CFI***	0.939			0.920			0.935		
***TLI***	0.928			0.905			0.922		
***SRMR***	0.061			0.075			0.062		

S.L., Standardized loadings; CR, Composite reliability; AVE, Average variance extracted; CFI, Comparative fit index; TLI, Tucker-Lewis Index; SRMR, Standardized Root Mean Square Residual. ^***^p < 0.001.

### 4.2 Structural models

The structural model analysis reveals key relationships between perceived walkability, walking frequency, and BMI. Specifically, we found that perceived walkability positively influences walking frequency, indicating that individuals who view their neighborhoods as more walkable tend to walk more often. Conversely, walking frequency has a negative impact on BMI, suggesting that increased walking is associated with lower BMI levels (Table 3). In addition, the correlation between perceived walkability and BMI was investigated by calculating both direct and indirect impacts. The total indirect effect, which is −0.064, mediated by other variables is statistically significant, while the direct effect shows a non-significant *p*-value. These results partially confirmed Hypothesis 2, i.e., perceived walkability has a significant effect on the reduction of BMI, but only as it is mediated by the other latent variables.

**Table 3:** Results of structural models and hypothesis testing (all data).

Hypotheses	Path	Coefficients	S.E.	Supported?
H1	PW → WF	0.118^**^	0.047	Yes
H2a	PW → BMI (direct)	−0.044	0.045	Partially supported
H2a	PW → BMI (indirect)	−0.064^**^	0.029	Partially supported
H2b	WF → BMI	−0.518^***^	0.035	Yes
H3a	PW → SC	0.268^***^	0.053	Yes
H3b	SC → WF	0.134^***^	0.048	Yes
H3c	SC → BMI	−0.007	0.04	No
Control variable				
Age→PW		−0.131^**^	0.050	-
Income→PW		0.195^***^	0.050	-
Driver license→PW		0.111^*^	0.064	-
Age→WF		−0.322^***^	0.041	-
Income→WF		−0.15^***^	0.043	-
PT card→WF		0.171^***^	0.052	-
Age→BMI		0.098^**^	0.041	-
Marriage→BMI		0.090^**^	0.038	-
Age→SC		0.158^***^	0.050	-
Income→SC		−0.112^**^	0.049	-

S.E., Standard error, ^*^p < 0.1, ^**^p < 0.05, ^***^p < 0.01.

It is important to highlight the role of social cohesion in the above interrelations. According to the empirical results in Table 3, when individuals perceive their environment as more walkable, they are more likely to engage in social interactions and build connections within their community. And the enhanced social cohesion encourages residents to walk more frequently. However, the path between social cohesion and BMI is not supported by the model results for the full sample.

The model also controlled for five socio-demographic variables, only significant correlations are shown in Table 3. The results show that both the evaluation of perceived walkability, social cohesion, walking frequency, and BMI are significantly related to age. Specifically, as age increases, there is a decrease in the perception of walkability and in the frequency of walking. This could be due to age-related declines in health functions, which present more challenges for engaging in physical activities, including walking. Concurrently, as age advances, there is an observed increase in the sense of social cohesion and BMI, which may reflect the tendency for older individuals to engage more with their communities and experience weight gain commonly associated with older age.

In addition, income is significantly positively related to perceived walkability, while it imposes negative effects on social cohesion and waking frequency. The positive effects of marriage on BMI have been confirmed in our model, it is plausible that marriage promotes opportunities for eating because married people are more inclined to eat together with their spouses and thus increase their intake (48).

The ownership of public transportation cards contributes to walking; it is possible because walking to bus stops increases walking frequency when using public transportation (16). The ownership of a driver license has a positive connection with perceived walkability.

### 4.3 Multiple-group analysis

After SEM analysis of the whole sample, we measured the structural invariance across gender of older adults by multiple-group analysis, and the results are shown in Table 4. A multiple-group analysis was performed hierarchically by restricting parameters. The chi-square difference tests enabled us to identify whether the invariance of nested models holds. First, the chi-square differences test indicates that the freely estimated Model 1 (*configural invariance*) and Model 2 (*weak invariance*) are invariant, i.e., the factor loadings of related variables are the same for both male and female older adults. Next, by comparing Model 2 and Model 3 (Table 4), which further imposed equality constraints on both the factor loadings and the intercepts, chi-square differences test results also show that invariability exists across different groups. However, the chi-square differences test results of the comparison of Model 3 and Model 4 (*strict invariance*) are significant, which supports that there is a considerable difference between male and female older adults.

**Table 4:** Measurement invariance.

Model specification	*χ*²	df	RMSEA	CFI	TLI	*p* value	Nested
(1) Configural invariance	799.84	438	0.056	0.929	0.913		
(2) Weak invariance	814.74	450	0.055	0.929	0.915	0.247	(2)–(1)
(3) Strong invariance	836.09	467	0.055	0.927	0.917	0.211	(3)–(2)
(4) Strict invariance	886.38	484	0.056	0.921	0.913	0.000	(4)–(3)

χ^2^, Model chi-square test; CFI, Comparative fit index; TLI, Tucker-Lewis index; RMSEA, Root Mean Square Error of Approximation.

To analyze gender differences in the relationships among perceived walkability, social cohesion, walking frequency, and BMI, we performed the Wald test to examine the significant differences in pathways between male older adults and female older adults. Table 5 provides a comprehensive illustration of such differences across the two groups. According to the results of the Wald test, the most significant gender gaps occur in the path between social cohesion between BMI.

**Table 5:** Structural model results of male and female model.

Hypotheses	Path	Male (*N* = 227)	Female (*N* = 306)	Difference
H1	PW → WF	0.078	0.149^**^	NS
H2a	PW → BMI (direct)	−0.058	−0.072	NS
H2a	PW → BMI (indirect)	−0.131	−0.053^*^	NS
H2b	WF → BMI	−0.559^***^	−0.511^***^	NS
H3a	PW → SC	0.225^***^	0.325^***^	NS
H3b	SC → WF	0.142^*^	0.103^*^	NS
H3c	SC → BMI	−0.083^*^	0.069	^**^
Control variable				
Age→PW		−0.187^***^	−0.063	NS
Income→PW		0.266^***^	0.115^*^	NS
Driver license→PW		−0.190^*^	0.145^*^	^**^
Age→WF		−0.390^***^	−0.216^***^	NS
Income→WF		−0.125^*^	−0.187^***^	NS
PT card→WF		0.185^**^	0.166^***^	NS
Driver license→WF		0.058	−0.113^*^	NS
Age→BMI		0.152^***^	−0.017	NS
Marriage→BMI		0.099^*^	0.118^**^	NS
Age→SC		0.197^**^	0.069	NS
Income→SC		−0.122^*^	0.073	NS
Marriage→SC		−0.141^**^	−0.095	NS

^*^p < 0.1, ^**^p < 0.05, ^***^p < 0.01; NS, Not significant.

In terms of the role of perceived walkability on walking frequency and BMI (Table 5), the effect of perceived walkability on walking frequency is mixed in different groups, a more positive evaluation of walkability increases the walking frequency for female older adults only with a standardized coefficient of 0.149, there is no significant correlation for their male counterparts. However, the data does not support the relationships between perceived walkability and BMI for both two models suggesting that they are not directly associated. Indirect effects were further calculated for both male and female older adults. It is interesting to note that the female model results are in line with the full sample model indicating that perceived walkability is indirectly related to BMI mediating by the other latent constructs with a correlation of −0.053, which partially supported our initial hypothesis. Significant indirect correlations, however, are not found in male models.

As shown in Table 5, as the frequency of walking increases, BMI to decrease among both male and female older adults, the impact on them is basically the same because their correlations are −0.559 and −0.511, respectively. This suggests that regular walking can be an effective strategy for weight management and potentially reducing obesity risks in older adult populations.

Social cohesion indeed has some effects on the correlations between perceived walkability, walking frequency, and BMI. As expected, the effect of perceived walkability is highly significant and positively related to the social cohesion for both males (0.225) and females (0.325), with a higher impact on female older adults. That is, better walkability encourages more walking trips, which is in accordance with previous research (33). The effects of social cohesion and walking frequency are positively significant for both male (0.142) and female (0.103) seniors. Considering the magnitude of the coefficients, the relationships of male older adults are stronger than that of female older adults. A mixed finding is found when measuring relationship between social cohesion and BMI. The negative effects of social cohesion on BMI are only confirmed for male older adults with a standardized coefficient of −0.083, while no significant effect is shown in the female model.

Only significant socio-economic variables are shown in Table 5. A few of them are shown to have a statistically significant influence on either male and/or female models. For example, age is naturally to be found to be negatively and significantly associated with walking frequency for both male and female older adults, suggesting that walking frequency tends to decrease with increasing age. Besides, as male seniors age, their evaluation of the walkability of their neighborhood becomes less positive, possibly due to reduced mobility or changes in their perception of the environment. It is also noteworthy that age is significantly positively related to social cohesion for the male model.

The income variable is also an important determinant that differed significantly depending on gender. Both males and females with higher incomes have a more positive evaluation of perceived walkability and less walking frequency. And it is positively associated with social cohesion only in the male group.

Married women are connected with less walking frequency but an elevated BMI. Marriage is linked with higher values of BMI, which is consistent with the results of the full sample model. Besides, married male older adults tend to have a less positive feeling of social cohesion; it is probably because they are more willing to participate in activities with their spouse, thus reducing the opportunities to interact with people in the same neighborhood.

The ownership of public transportation cards contributes to walking for male older adults, which is also in accordance with the full sample model. The effects of owning a driver license are adverse in the male model and female model. They are negatively related to male older adults, while they are positively related to female older adults. Besides, female older adults who own a driver license tend to have less walking frequency.

## 5 Discussion

In the pursuit of healthier and more age-friendly cities for both male and female older adults, it is essential to improve the understanding of whether transportation-related elements, such as neighborhood walkability, neighborhood social environment, and walking frequency, would help reduce increasingly common obesity issues and distinguish whether those relationships would differ by gender. Hence, a Structural Equation Model (SEM) and multiple-group analysis were applied to answer these questions.

Our findings confirm that gender gaps exist in correlations between perceived walkability, social cohesion, and walking frequency on how BMI is influenced. The most substantial difference exists in the effects of social cohesion on BMI.

Our results provide empirical support for Hypothesis 1 for the full sample model, indicating a positive association between perceived walkability and walking frequency. We can conclude that an increase in perceived walkability is linked to a higher frequency of walking among the older adult population. The effect of perceived walkability on walking frequency is mixed in different groups, a more positive evaluation of walkability increases the walking frequency for female older adults only, there is no significant correlation for their male counterparts. Perhaps women walk more when they feel more comfortable and other people offer them a socially safe walking environment by providing “eyes on the street” (49). Similarly, women tend to participate in more out-of-home travel related to family affairs, thus their perceptions of access to destinations like grocery stores and other shops might be more influential for walking for women older adults than their male counterparts (50).

Both perceived walkability and walking contribute to the reduction of BMI to some extent. To be specific, for the full sample model, Hypothesis 2, which proposed a direct negative correlation between perceived walkability and BMI, was only partially supported. This indicates that the positive perception of walkability contributes to lower BMI indirectly by encouraging more frequent walking. However, Hypothesis 3, older adults who have more walking frequencies have a lower BMI is supported. For male and female models, the promoting effects of perceived walkability on walking are only substantiated for female older adults, while there is no significant connection exists in the male model, which is incongruous with previous studies that a general positive correlation between them for both male and female older adults (11, 16). Perhaps women walk more when they feel more comfortable and other people offer them a socially safe walking environment by providing “eyes on the street” (49). Similarly, women tend to participate in more out-of-home travel related to family affairs, thus their perceptions of access to destinations like grocery stores and other shops might be more influential for walking for women older adults than their male counterparts (50). Besides, only indirect negative effects of perceived walkability on BMI are found for female older adults. It is also worth noting that the alleviating effects of walking on BMI are demonstrated in both male and female models indicating that the more walking trips, the lower weight for older adults. Bodyweight gain is usually caused by gaps between energy intake and consumption (7). Moderate physical activity like walking might help burn more calories than those who do not participate in such activity, which is in line with previous studies (8, 13).

Intriguingly, the effects of social cohesion stand out. Both the positive relationships between perceived walkability and social cohesion and the promoting effects of social cohesion on the walking frequency of the two models confirm our initial hypotheses (H3a and H3b). The former connection, however, is stronger for female older adults than their male counterparts, while the strength of the latter association is slightly larger for male seniors. The possible reason is that a better walking environment draws seniors out into the neighborhood, which leads to greater familiarity and social cohesion. Besides, older adults may feel safer and more comfortable walking in a familiar neighborhood where they know they can ask for help if there is a problem. The negative connection between social cohesion and BMI is only verified in the male model.

Several limitations of this study are worth mentioning, which could be further addressed in future studies. First, constrained by the available data, only correlation rather than causation between perceived walkability, social cohesion, walking frequency, and BMI could be made. The exploration of cause-effect relationships using longitudinal data is needed for the causalities will help yield more affirmative conclusions when detecting such kinds of relationships. In this way, it might provide more strong evidence for policy implications. Second, only walking was chosen for this study. Additional physical activities of different intensities can also be incorporated into future studies. For example, activities at moderate or greater intensity like jogging, dancing, bicycling, etc. contribute to the BMI of older adults in a different way. Finally, although perceptions of the built environment are considered good indicators to represent their awareness of residential neighborhoods and are widely used in studies on health (51), the debate on whether the objective or perceived built environment variables should be used remains inconclusive. It would be of great interest for future research to build models considering both objective and perceived measures of the built environment.

## 6 Conclusion

The findings of our study have potential implications that might be valuable to improving the health status and health-related travel behavior of older adults. Our findings also provide a general reference to other second-tier cities similar to Dalian. First, in order to achieve a healthy and inclusive environment to promote older adults’ health in general, it is important to pay special attention to gender gaps, because women older adults are in a more vulnerable situation compared to their male counterparts. Policymakers always believe that any intervention that can improve health overall will be suitable for older adults and address the gender difference (2, 16). However, policies targeting the unique needs of different gender will be more efficient. For instance, various incentive interventions should be implemented for different gender. Our models indicate that female older adult is more sensitive to perceived walkability. Thus, the improvement of walkability is important for them to induce more walking and lose weight. The promotion of an objective built environment will help enhance the perceived walkability. For example, the majority of older adults in China live in relatively old neighborhoods. Old neighborhoods refer to those built before 2000 with low construction standards, incomplete amenities, aging infrastructure, backward public facilities, etc., which in turn affect the basic life and quality of life of residents. Government and policymakers should primarily focus on renovating the steep steps and uneven roads in those neighborhoods, which will alleviate the fears of walking and ameliorate the perception of walkability. On the other hand, the effects of social cohesion seem more important to male older adults. Hence, the improvement of the social environment might be more efficient for them to improve health-related and prosocial activity. Turning vacant buildings in those old neighborhoods into community centers, senior centers, etc. could help seniors to meet more people who live in the same community, which will make them feel more socially integrated. This stronger sense of community might make them more satisfied with their neighborhood and induce more walking trips.

## Data availability statement

The original contributions presented in the study are included in the article/Supplementary material, further inquiries can be directed to the corresponding author/s.

## Ethics statement

Ethical review and approval was not required for the study on human participants in accordance with the local legislation and institutional requirements. Written informed consent from the patients/ participants or the patients’/participants’ legal guardian/next of kin was not required to participate in this study in accordance with the national legislation and the institutional requirements.

## Author contributions

JM: Conceptualization, Formal analysis, Investigation, Methodology, Software, Validation, Visualization, Writing – original draft, Writing – review & editing. WL: Formal analysis, Investigation, Methodology, Resources, Validation, Visualization, Writing – review & editing.
