# Supplementary material for: Exploring the group heterogeneity in the impact of social cohesion on the walking frequency of older adults in China
Source: Front Public Health. 2024 Jul 31;12:1424975. doi: 10.3389/fpubh.2024.1424975 (PMC11322150; doi:10.3389/fpubh.2024.1424975)
Supplement: Supplementary file 1 [file Table_1.pdf]

Survey on walking behavior of the older adult

Dear Respondent,

Greetings! We are very grateful for your cooperation in this survey.

This study aims to anonymously investigate the close relationship between walkable neighborhoods and the increase in walking frequency, as well as the strengthening of social cohesion. Our objective is to analyze how these factors interact with each other to contribute to the reduction of Body Mass Index (BMI) and other positive health-related outcomes. Through this research, we hope to provide scientific evidence and strategic recommendations for enhancing the quality of life for residents and promoting the development of healthy communities.

We are committed to maintaining the strictest confidentiality of your responses. All data collected will be securely stored and anonymized to ensure that no individual can be identified from the information provided. We have implemented stringent data protection measures to safeguard your privacy.

We thank you once again for considering to take part in this study. Your insights are invaluable, and we assure you that the results will contribute to a broader understanding of how urban design can foster healthier lifestyles. Please feel free to ask any questions or express any concerns you may have. Your cooperation is greatly appreciated, and we look forward to your valuable input.

Questions:

1. How many times did you walk for transportation in the past week?

2. The term "residential area" refers to the daily life range that is accessible within a 10–15-minute walk from your residence.

Please select your responses to the following questions based on your level of agreement with the current state of the environment in the residential area where you live.

	Description	Strongly Disagree	Disagree	Neutral	Agree	Strongly Agree
**Perceived Walkability**						
Accessibility	It is easy to walk from my house to the shops.					
	It is easy to walk to a transit stop from my home.					
	It is easy to go to various places from my house in my neighborhood.					
Road Condition	The road around my house is in good condition in terms of greenery.					
	The roads are clean and tidy in my neighborhood.					
	My neighborhood streets are well lit at night.					
	The streets in my neighborhood are flat.					
Aesthetics	There are enough places around my house exercise.					
	The natural environment around my home is good, which makes me want to go out and walk.					
	The buildings around my house are very attractive.					
Safety	My neighborhood is very safe.					
	My neighborhood is very safe during the day.					
	My neighborhood is very safe at night.					
**Social Cohesion**						
Mutual help	Residents in my neighborhood are willing to help their neighbors					
Connection	I live in a tight-knit community.					
Trust	The residents in this community are trustworthy.					
Acquaintanceship	I know most of my neighbors.					

3.Gender: □ Female □ Male

4.Date of birth is: ________ year ________ month

5.Height is ________ centimeters, weight is ________ kilograms

6.Marital status is: □ Single □ Married and spouse is alive □ Divorced □ Widowed

7.Household annual income situation:

□ Below 50,000 yuan □ 50,000-100,000 yuan □ 100,000-150,000 yuan □ 150,000-200,000 yuan □ Above 200,000 yuan

8.The transportation cards/certificates you have (multiple choices allowed):

□ Car driver's license □ Public transportation card (□ Yes □ No for senior citizen discount card)
